# Supplementary material for: Exposure to landscape fire smoke reduced birthweight in low- and middle-income countries: findings from a siblings-matched case-control study
Source: eLife. 2021 Sep 29;10:e69298. doi: 10.7554/eLife.69298 (PMC8563002; doi:10.7554/eLife.69298)
Supplement: Source code 1. [file elife-69298-supp2.zip › R-codes.html]

Exposure to landscape fire smoke extremely reduced birthweight in low- and middle-income countries: findings from a siblings-matched case-control study


# Exposure to landscape fire smoke extremely reduced birthweight in low- and middle-income countries: findings from a siblings-matched case-control study

#### Jiajianghui Li; Tianjia Guan; Qian Guo; Guannan Geng; Huiyu Wang; Fuyu Guo; Jiwei Li; Tao Xue

#### 2021/5/5

## Introduction

This file documents the R codes for reproduce the figures in the manuscript titled as “Exposure to landscape fire smoke extremely reduced birthweight in low- and middle-income countries: findings from a siblings-matched case-control study”.

We also provide the raw R codes for our epidemiological models.

### Figure 1

The following R code can be used to reproduce Figure 1. However, since the geographic locations of DHS data are owned by the third parity, they are removed from the following source data. Those geographic locations should be directly and freely obtained from https://dhsprogram.com/ after a registration.

The source data contains three datasets:

1. fire\_map: the background map of average fire-sourced PM2.5
2. geo\_rate: the location of surveyed countries and the rate of case
3. world\_map: the world map of country-level boundaries.

```
library(ggplot2)
```

```
## Warning: package 'ggplot2' was built under R version 4.0.4
```

```
library(ggsci)
load("Fig 1 source data.RData")
ggplot(data=fire_map)+
  geom_tile(aes(x=lon,y=lat,fill=val))+
  scale_fill_material("deep-orange",name=expression(atop('Fire-sourced',PM[2.5]~(mu~g/m^3))),breaks=seq(5,30,5))+
  geom_polygon(data=world_map,aes(x=long,y=lat,group=group),col="grey70",fill=NA,alpha=0,size=0.3)+
  theme_void()+
  geom_point(data=geo_rate,aes(x=lon,y=lat,size=val),shape=1,col="navy")+
  scale_size(breaks=c(0,5,10,20,30),labels=c("5 -","5 ~ 10","10 ~ 20","20 ~ 30","30 +"),name="Rate (per 1000) of\nvery low birthweight (< 1500 g)\namong the analyzed samples")+
  scale_x_continuous(expand=c(0,0))+
  scale_y_continuous(limits=c(-60,84))+
  theme(legend.position=c(0.01,0.01),legend.justification =c(0,0),legend.box = "vertical")+
  guides(size=guide_legend(override.aes = list(shape=1),order = 3),
         fill=guide_legend(order=2,keyheight = unit(0.03,"npc")))
```

```
## Loading required package: sp
```

```
## Regions defined for each Polygons
```

### Figure 2

The following R code can be used to reproduce Figure 2. The source data contain a dataset named coef, which documents all estimates from different regression models.

```
library(ggplot2)
load("Fig 2 source data.RData")
ggplot(data=coef)+
  geom_linerange(aes(x=lag2,ymin=lo,ymax=up,group=lag),position=position_dodge(width=0.75))+
  geom_point(aes(x=lag2,y=mid,group=lag,shape=lag,col=sig),position=position_dodge(width=0.75))+
  facet_wrap(~y,scales="free_x",strip.position = "bottom",nrow=1)+
  coord_flip()+
  theme_minimal()+
  ylab(expression("Estimate for per increment of"~1~mu~g/m^3~'in exposure to fire-sourced'~PM[2.5]~'before birth'))+
  geom_hline(aes(yintercept=0),linetype=2,col=1)+
  theme(axis.text.x=element_text(vjust=0.5),
        strip.background  = element_rect(fill="grey90",color=NA),
        legend.position = "top",legend.direction = "horizontal")+
  scale_x_discrete(labels=parse(text=levels(coef$lag2)))+
  xlab("Sequentially adjusted models")+
  scale_shape_manual(values=c(4,15,16),name="Exposure time-window (month)")+
  scale_color_manual(values=c("black","red"),name="P-value < 0.05")
```

### Figure 3

The following R code can be used to reproduce Figure 3.

The source data contain two datasets:

1. curve: the nonlinear association between fire-sourced PM2.5 and birthweight change
2. exposure: the summary statistics for the exposure data

```
library(ggplot2)
load("Fig 3 source data.RData")
ggplot(data=curve)+
  geom_path(aes(x=x,y=lo),linetype=2)+
  geom_path(aes(x=x,y=up),linetype=2)+
  geom_path(aes(x=x,y=fit))+
  geom_rect(data=exposure,aes(xmin=lo,xmax=up,ymin=y,ymax=y),col=1)+
  geom_rect(data=exposure,aes(xmin=q25,xmax=q75,ymin=y-2,ymax=y+2),fill="white",col=1)+
  geom_rect(data=exposure,aes(xmin=q50,xmax=q50,ymin=y-2,ymax=y+2),fill="white",col=1)+
  geom_point(data=exposure,aes(x=mu,y=y),col=2,size=1)+
  scale_fill_npg()+
  scale_color_npg()+
  theme_bw()+
  xlab(expression('Level of gestational exposure to fire-sourced'~PM[2.5]~(mu~g/m^3)))+
  geom_hline(aes(yintercept=0),linetype=1,col="grey50")+
  theme(legend.position = c(0.99,0.99),
        legend.justification = c(1,1),
        legend.title = element_blank(),
        strip.placement = "outside",
        panel.grid.major.x = element_blank(),
        panel.grid.minor.y = element_blank(),
        panel.grid.minor.x = element_blank(),
        axis.title.y=element_text(hjust=0.3))+
  geom_text(data=exposure,aes(x=up,y=y,label=reg),hjust=-0.1,vjust=0.5,size=5)+
  scale_y_continuous(breaks=c(0,seq(-150,-25,25)))+
  scale_x_continuous(limits=c(0,40))+
  ylab("Birthweight change (g)")
```

### Figure 4

The following R code can be used to reproduce Figure 4.

The source data contain two datasets:

1. curve: the effect of fire-sourced PM2.5 varying with mean birthweight
2. outcome: the summary statistics for the birthweight

```
library(ggplot2)
load("Fig 4 source data.RData")
ggplot(data=curve)+
  geom_path(aes(x=x,y=lo),linetype=2)+
  geom_path(aes(x=x,y=up),linetype=2)+
  geom_path(aes(x=x,y=fit))+
  geom_rect(data=outcome,aes(xmin=lo,xmax=up,ymin=y,ymax=y),col=1)+
  geom_rect(data=outcome,aes(xmin=q25,xmax=q75,ymin=y-2,ymax=y+2),fill="white",col=1)+
  geom_rect(data=outcome,aes(xmin=q50,xmax=q50,ymin=y-2,ymax=y+2),fill="white",col=1)+
  geom_point(data=outcome,aes(x=mu,y=y),col=2)+
  scale_fill_npg()+
  scale_color_npg()+
  theme_bw()+
  xlab("Birthweight (g)")+
  geom_hline(aes(yintercept=0),linetype=1,col="grey50")+
  theme(legend.position = c(0.99,0.99),
        legend.justification = c(1,1),
        legend.title = element_blank(),
        strip.placement = "outside",
        panel.grid.major.x = element_blank(),
        panel.grid.minor.y = element_blank(),
        panel.grid.minor.x = element_blank(),
        axis.title.y=element_text(hjust=0))+
  geom_text(data=outcome,aes(x=mu,y=y-2,label=reg),hjust=0.5,vjust=1.1,size=5)+
  scale_y_continuous(breaks=c(0,seq(-100,-20,20)),labels=c(0,seq(-100,-20,20))/10)+
  ylab(expression("Relative birthweight change (%) per 1"~mu~g/m^3~"increment in fire-sourced"~PM[2.5]))+
  scale_x_continuous(limits=c(1000,6000),breaks=seq(1000,6500,500))
```

### Main epidemiological models

The raw R codes for customized epidemiological models are documented as follows. However, those codes are provided for transparency purpose. Because we are not permitted to re-distribute the DHS data, owned by the third party, the inputs for the R codes are not provided here. To reproduce those results, the R codes should be performed with inputted data downloaded from DHS website (https://dhsprogram.com/), and prepared as we described in the manuscript.

#### Linear models with different settings

The ‘Regression Inputs.RData’ documents five datasets involved into the epidemiological models that link environmental exposures with birthweight.

1. lng: individual records on birthweight and relevant covarites, including

- ID: ID of a mother
- birth.order: birth order for a specific child of a mother
- multiple: singleton birth or not
- child.sex: sex of a child
- maternal.age: maternal age for a specific child of a mother
- birth.month: calendar month (1-12) of the birthday
- birth.year: calendar year of the birthday
- cc: country ID

2. PM25F\_g10: a matrix to record monthly exposures to fire-sourced PM2.5 for the month of birth and preceding 9 months; one row is for a child;
3. PM25m\_g10: the similar exposure matrix for total PM2.5;
4. TMP\_g10: the similar exposure matrix for temperature;
5. HTY\_g10: the similar exposure matrix for humidity.

```
load("Regression Inputs.RData")
library(fixest)
fs<-c("y~PM25F|ID",
      "y~PM25F+ns(TMP,3)+ns(HTY,3)|ID",
      "y~PM25F+ns(TMP,3)+ns(HTY,3)+as.factor(birth.order)+as.factor(multiple)+as.factor(child.sex)+ns(maternalage,3)|ID",
      "y~PM25F+ns(TMP,3)+ns(HTY,3)+as.factor(birth.order)+as.factor(multiple)+as.factor(child.sex)+ns(maternalage,3)+I(PM25m-PM25F)|ID",
      "y~PM25F+ns(TMP,3)+ns(HTY,3)+as.factor(birth.order)+as.factor(multiple)+as.factor(child.sex)+ns(maternalage,3)+ns(birth.month,4)+ns(birth.year,5)+I(PM25m-PM25F)|ID",
      "y~PM25F+ns(TMP,3)+ns(HTY,3)+as.factor(birth.order)+as.factor(multiple)+as.factor(child.sex)+ns(maternalage,3)+ns(birth.month,4)+ns(birth.year,5)+cc:birth.year+I(PM25m-PM25F)|ID"
)
names(fs)=c("unadjusted","climate","demographic","PM25","trend","spacetime")
coef=NULL

for(lag in 1:3)
{
  lng$PM25F=apply(PM25F_g10[,1:(lag*3)],1,mean)
  lng$PM25m=apply(PM25m_g10[,1:(lag*3)],1,mean)
  lng$TMP=apply(TMP_g10[,1:(lag*3)],1,mean)
  lng$HTY=apply(HTY_g10[,1:(lag*3)],1,mean)
  
  for(f in fs)
  {
    m<-feols(as.formula(f),data=lng[id,])
    tmpr=rbind(cbind(as.data.frame(m$coeftable)[1,]))
    tmpr$model=names(which(fs==f))
    tmpr$lag=lag
    
    coef=rbind(coef,tmpr)
  }
}
```

#### Nonlinear model for exposure-response function between birthweight and fire-sourced PM2.5

We estimate the nonlinear exposure-response function using a thin plate spline adapted from the mgcv package. The nonlinear association is estimated by the fully-adjusted model, and is coded as ‘nonlinear\_model’.

```
library(mgcv)
b<-gam(y~s(PM25F)-1,data=lng)
bs=model.matrix(b)
colnames(bs)=paste("bs",1:dim(bs)[2],sep="")
nonlinear_model<-feols(as.formula(gsub("~PM25F",paste("~",paste(colnames(bs),collapse = "+"),sep=""),fs[6])),data=cbind(bs,lng))
```

#### Baseline-varying association model

The baseline-varying association is estimated by the fully-adjusted model, and is coded as ‘baseline\_varying\_model’.

We first calculate the mean birthweight for each group, in which children are affiliated to the same mother.

```
tmp=aggregate(y~ID,data=lng,mean,na.rm=T)
rownames(tmp)=tmp$ID
lng$mu=tmp[as.character(lng$ID),"y"]
lng$y=lng$y-lng$mu
```

The baseline-varying association model is customized from the ‘varying coefficient model’ provided by the mgcv package. The model is coded as ‘baseline\_varying\_model’.

```
b<-gam(y~s(mu,by=PM25F)-1,data=lng)
vbs=model.matrix(b)
colnames(vbs)=paste("vbs",1:dim(vbs)[2],sep="")
baseline_varying_model<-feols(as.formula(gsub("~PM25F",paste("~",paste(colnames(vbs),collapse = "+"),sep=""),fs[6])),data=cbind(vbs,lng))
```
