# Supplementary material for: Exposure to landscape fire smoke reduced birthweight in low- and middle-income countries: findings from a siblings-matched case-control study
Source: eLife. 2021 Sep 29;10:e69298. doi: 10.7554/eLife.69298 (PMC8563002; doi:10.7554/eLife.69298)
Supplement: Transparent reporting form [file elife-69298-transrepform1.docx]

***eLife’s* transparent reporting form**

We encourage authors to provide detailed information *within their submission* to facilitate the interpretation and replication of experiments. Authors can upload supporting documentation to indicate the use of appropriate reporting guidelines for health-related research (see [EQUATOR Network](http://www.equator-network.org/%20)), life science research (see the [BioSharing Information Resource](https://biosharing.org/)), or the [ARRIVE guidelines](http://www.plosbiology.org/article/info:doi/10.1371/journal.pbio.1000412) for reporting work involving animal research. Where applicable, authors should refer to any relevant reporting standards documents in this form.

If you have any questions, please consult our Journal Policies and/or contact us: [editorial@elifesciences.org](mailto:editorial@elifesciences.org).

**Sample-size estimation**

- You should state whether an appropriate sample size was computed when the study was being designed
- You should state the statistical method of sample size computation and any required assumptions
- If no explicit power analysis was used, you should describe how you decided what sample (replicate) size (number) to use

Please outline where this information can be found within the submission (e.g., sections or figure legends), or explain why this information doesn’t apply to your submission:

•	You should state whether an appropriate sample size was computed when the study was being designed

NA

•	You should state the statistical method of sample size computation and any required assumptions

NA

•	If no explicit power analysis was used, you should describe how you decided what sample (replicate) size (number) to use

This is an observational study, which utilized all available samples meeting our inclusion criteria from all available DHS surveys when conducting this study. The study period 2000-2014 is determined by the limited availability of exposure data.

**Replicates**

- You should report how often each experiment was performed
- You should include a definition of biological versus technical replication
- The data obtained should be provided and sufficient information should be provided to indicate the number of independent biological and/or technical replicates
- If you encountered any outliers, you should describe how these were handled
- Criteria for exclusion/inclusion of data should be clearly stated
- High-throughput sequence data should be uploaded before submission, with a private link for reviewers provided (these are available from both GEO and ArrayExpress)

Please outline where this information can be found within the submission (e.g., sections or figure legends), or explain why this information doesn’t apply to your submission:

•	You should report how often each experiment was performed

NA, this is an observational study. Therefore, no replicate is available.

•	You should include a definition of biological versus technical replication

NA

•	The data obtained should be provided and sufficient information should be provided to indicate the number of independent biological and/or technical replicates

Yes. The relevant statement reads as “*Finally, our dataset assembled 113 individual surveys from 54 countries (Figure 1) and included 227,948 newborns classified into 109,137 groups according to their mothers.*”.

•	If you encountered any outliers, you should describe how these were handled

NA. No outlier is excluded.

•	Criteria for exclusion/inclusion of data should be clearly stated

Yes. The relevant statement reads as “We extracted the females with at as least two valid records of newborns from all available individual DHSs. Each valid record was defined using the following inclusion criteria: (1) a valid birthweight, (2) a record of the GPS coordinates, (3) a valid birthdate, and (4) valid environmental exposure values (some of the satellite-based PM_2.5_ estimates were not available for some locations, such as small islands).”

•	High-throughput sequence data should be uploaded before submission, with a private link for reviewers provided (these are available from both GEO and ArrayExpress)

NA

**Statistical reporting**

- Statistical analysis methods should be described and justified
- Raw data should be presented in figures whenever informative to do so (typically when N per group is less than 10)
- For each experiment, you should identify the statistical tests used, exact values of N, definitions of center, methods of multiple test correction, and dispersion and precision measures (e.g., mean, median, SD, SEM, confidence intervals; and, for the major substantive results, a measure of effect size (e.g., Pearson's r, Cohen's d)
- Report exact p-values wherever possible alongside the summary statistics and 95% confidence intervals. These should be reported for all key questions and not only when the p-value is less than 0.05.

Please outline where this information can be found within the submission (e.g., sections or figure legends), or explain why this information doesn’t apply to your submission:

•	Statistical analysis methods should be described and justified

A fixed-effect logit regression model is utilized to analyse the matched case-control data. The methods are documented in the subsection of *Statistical analyses*.

•	Raw data should be presented in figures whenever informative to do so (typically when N per group is less than 10)

NA. In all figures, samples are larger than 10.

•	For each experiment, you should identify the statistical tests used, exact values of N, definitions of center, methods of multiple test correction, and dispersion and precision measures (e.g., mean, median, SD, SEM, confidence intervals; and, for the major substantive results, a measure of effect size (e.g., Pearson's r, Cohen's d)

NA. This study is an observational study, not experimental study.

•	Report exact p-values wherever possible alongside the summary statistics and 95% confidence intervals. These should be reported for all key questions and not only when the p-value is less than 0.05.

All results are reported with 95% confidence intervals.

(For large datasets, or papers with a very large number of statistical tests, you may upload a single table file with tests, Ns, etc., with reference to sections in the manuscript.)

**Group allocation**

- Indicate how samples were allocated into experimental groups (in the case of clinical studies, please specify allocation to treatment method); if randomization was used, please also state if restricted randomization was applied
- Indicate if masking was used during group allocation, data collection and/or data analysis

Please outline where this information can be found within the submission (e.g., sections or figure legends), or explain why this information doesn’t apply to your submission:

•	Indicate how samples were allocated into experimental groups (in the case of clinical studies, please specify allocation to treatment method); if randomization was used, please also state if restricted randomization was applied

NA. This is an observational study. We utilized all available data. No randomization has been applied.

•	Indicate if masking was used during group allocation, data collection and/or data analysis

NA. As an observational study, no masking has been applied.

**Additional data files (“source data”)**

- We encourage you to upload relevant additional data files, such as numerical data that are represented as a graph in a figure, or as a summary table
- Where provided, these should be in the most useful format, and they can be uploaded as “Source data” files linked to a main figure or table
- Include model definition files including the full list of parameters used
- Include code used for data analysis (e.g., R, MatLab)
- Avoid stating that data files are “available upon request”

Please indicate the figures or tables for which source data files have been provided:

•	We encourage you to upload relevant additional data files, such as numerical data that are represented as a graph in a figure, or as a summary table

All data embedded in figures have been submitted as .RData files.

•	Where provided, these should be in the most useful format, and they can be uploaded as “Source data” files linked to a main figure or table

The source data have been uploaded. The .RData files are preferred, because they can be directly linked to the R codes documented in supplemental materials.

•	Include model definition files including the full list of parameters used

The details on model specification are documented as R codes in the supplmental materials.

•	Include code used for data analysis (e.g., R, MatLab)

The R codes for data analyses are documented in the supplement.

•	Avoid stating that data files are “available upon request”

Confirmed. We state availability as “Source data for figures and R codes for the epidemiological models are documented in the supplemental file.”

Those files can be obtained from the supplemental file named as “R codes and source data.zip”.
